# Antiproliferative Effect and Ultrastructural Alterations Induced by Psilostachyin on *Trypanosoma cruzi*

**DOI:** 10.3390/molecules15010545

**Published:** 2010-01-25

**Authors:** Valeria Sülsen, Patricia Barrera, Liliana Muschietti, Virginia Martino, Miguel Sosa

**Affiliations:** 1 Cátedra de Farmacognosia, Universidad de Buenos Aires, Facultad de Farmacia y Bioquímica, IQUIMEFA (UBA-CONICET), Junín 956 (1113), CABA, Argentina; E-Mails: vsulsen@ffyb.uba.ar (V.S.); vmartino@ffyb.uba.ar (V.M.); 2 Facultad de Ciencias Médicas, Instituto de Histología y Embriología "Dr. Mario H. Burgos", Universidad Nacional de Cuyo-CONICET, CC 56 (5500), Mendoza, Argentina; E-Mails: patbarrera78@yahoo.com.ar (P.B.); msosa@fcm.uncu.edu.ar (M.S.)

**Keywords:** sesquiterpene lactone, psilostachyin, *Trypanosoma cruzi*, ultrastructural alterations

## Abstract

The effect of psilostachyin, a natural sesquiterpene lactone, on the growth and viability of cultured epimastigotes of *Trypanosoma cruzi* (Tulahuen) is reported. The antiproliferative effect was evaluated by counting the parasites in a Neubauer chamber and measuring their viability by using the dye exclusion technique. The effect on parasite growth was irreversible at concentrations higher than 1.0 µg/mL and the addition of glutathione only partially blocked the effect of the compound. Moreover, we have studied the effects of this natural compound on parasite ultrastructure by transmission electron microscopy. Interestingly, psilostachyin induced ultrastructural alterations on the parasites at a concentration of 0.5 µg/mL, with important mitochondrial swelling and deformity of the kinetoplast.

## 1. Introduction

Chagas disease or American trypanosomiasis is a major health problem in Latin America. It is estimated that some 18 million people are infected with *Trypanosoma cruzi*, the etiologic agent of Chagas disease, and 100 million people live in risk areas [1]. Unfortunately, the treatments used so far to alleviate this disease have not been successful. During the last 40 years only benznidazole and nifurtimox have been on the market and these drugs present many side effects and are poorly tolerated. Their use is restricted to the acute phase of the disease and long treatment courses are required [2,3]. For these reasons, the development of new safe and effective therapeutic agents is urgently needed.

Medicinal plants are a rich source of compounds with a wide range of chemical diversity and biological activities. In the field of antiprotozoal drugs, medicinal species have afforded several bioactive compounds [4,5,6,7]. Among them, sesquiterpene lactones (STLs) are terpenoid compounds, mainly from the Asteraceae family, for which many biological activities have been reported [8,9,10] including antiparasitic activity [11,12,13]. However, the specific targets for these compounds are poorly known.

We have recently reported the *in vitro* and *in vivo* trypanocidal activity against *Trypanosoma cruzi* (RA) of psilostachyin, a sesquiterpene lactone, isolated by bioassay-guided fractionation from *Ambrosia tenuifolia* [14]. *A. tenuifolia* Sprengel (Asteraceae) is an Argentine medicinal species popularly known as “altamisa”, “ajenjo” or “ajenjo del campo”, which is traditionally used to treat intermittent fevers and to eliminate intestinal worms [15].

As an approach in the search of molecular targets of STLs, the aim of this investigation was to evaluate the effect of psilostachyin on the proliferation and viability of cultured *T. cruzi* epimastigotes (Tulahuen) and at the ultrastructural features of the parasites.

## 2. Results and Discussion

### 2.1. Viability and reversibility assay

The trypanocidal activity of psilostachyin on *T. cruzi* epimastigotes and tripomastigotes (RA) has been previously reported (14). In order to go deep into the antiproliferative activity of this compound, two assays were carried on the non-infective form of *T. cruzi* (Tulahuen). Previous to these assays the effect of this compound on the growth of epimastigotes was measured. The STL exhibited an antiproliferative effect and inhibited parasite growth in a dose-dependent way. This effect was observable as early as 24 h. After 72 h of incubation, the STL induced a 58% growth inhibition at 0.5 µg/mL. The 50% inhibitory concentration (IC_50_) of psilostachyin was 0.3 µg/mL (1.1 µM), showing it to be more active than benznidazole (IC_50 _= 10.6 µM). 

**Figure 1:**
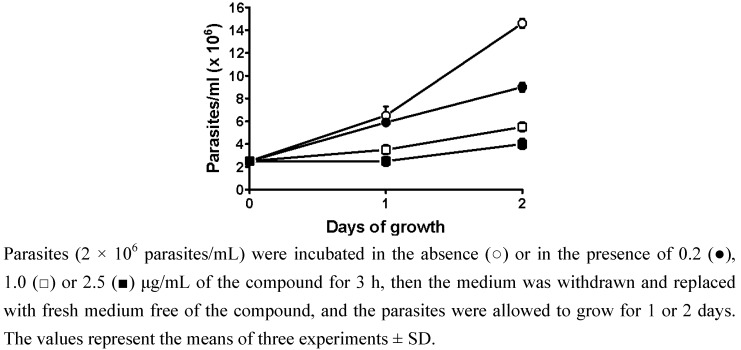
Irreversible effect of psilostachyin on the growth of *T. cruzi* epimastigotes.

The observed effect was completely irreversible at concentrations greater than 1.0 μg/mL and only partially reversible at concentrations as low as 0.2 μg/mL (Figure 1). DMSO (0.1%) alone, which was used as a solvent for the compound, showed no antiproliferative effect on the parasites (data not shown).

**Figure 2:**
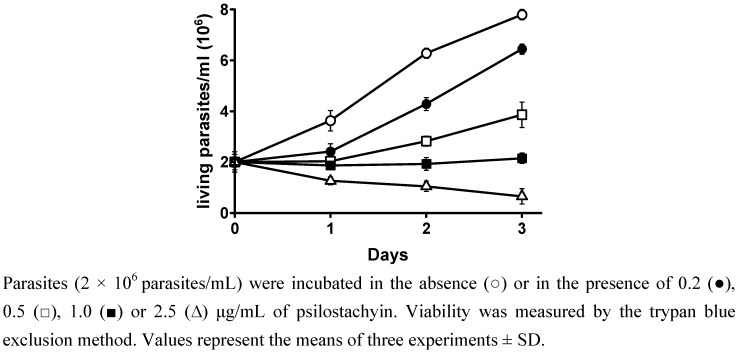
Effect of psilostachyin on the viability of *T. cruzi* epimastigotes.

Parasite viability was evaluated by the trypan blue exclusion method. High mortality of the parasites was observed as from the second day of incubation, when psilostachyin (2.5 μg/mL) was added to the culture (Figure 2).

These results indicated that the compound could act either as cytostatic or cytotoxic agent for the parasites, depending on the concentration and exposure time, and this effect was irreversible, since with psilostachyin the parasites did not recover even with few hours of incubation. 

### 2.2. Cytotoxicity assay on mammalian cells

The cytotoxicity of psilostachyin was assayed on murine T-lymphocytes. This STL showed a 50% cytotoxic concentration (CC_50_) of 3.0 μg/mL at 72 h. The selectivity index (SI) was calculated, in order to compare the trypanocidal activity and the toxicity for mammalian cells. The SI for psilostachyin was 10, thus indicating that the compound was more toxic to the parasite than to mammalian cells. 

### 2.3. Effect of psilostachyin on T. cruzi epimastigotes in the presence of glutathione (GSH)

Most STLs contain a common functional α-methylene-γ-lactone structure, which is highly reactive with thiol groups of proteins [13] and may thus block key enzymes for parasite growth and survival. In order to verify if this structure is responsible for the trypanocidal activity of psilostachyin, the effect of this compound in the presence of gluthatione (GSH) was evaluated.

**Figure 3:**
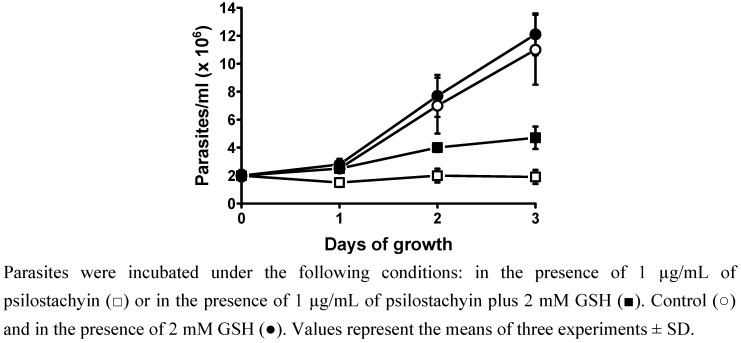
Effect of psilostachyin on the growth of *T. cruzi* in the presence of GSH.

As shown in Figure 3, the presence of GSH only partially blocked the compound’s effects, thus indicating that the α-methylene-γ-lactone by itself is not solely responsible for the activity of psilostachyin. 

### 2.4. Transmission electron microscopy

**Figure 4:**
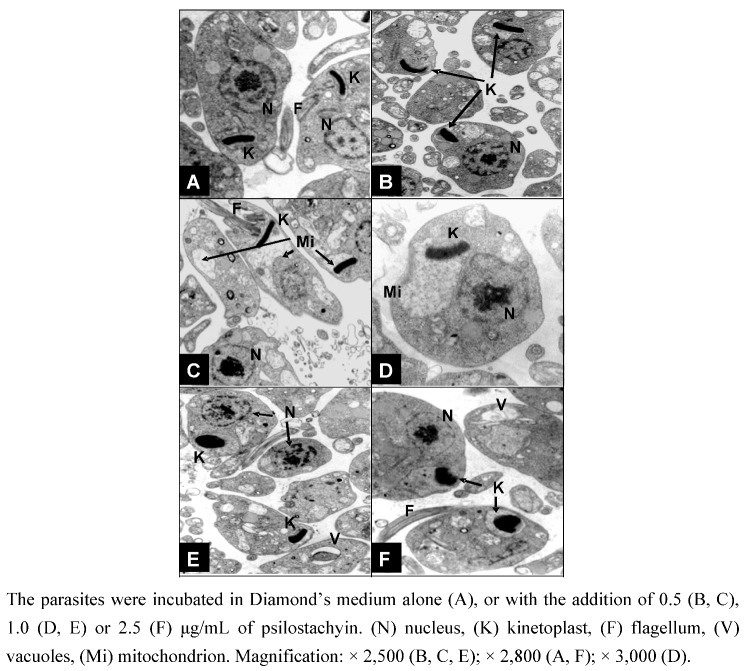
Effect of psilostachyin on ultrastructure of *T. cruzi* epimastigotes.

Ultrathin sections of parasite cells treated with different concentrations of psilostachyin (0.5–2.5 μg/mL) were observed with a transmission electron microscope (Figure 4). Psilostachyin induced significant alterations in the parasites, such as cytoplasmic vacuolization, a slight increase in multivesicular bodies and especially, mitochondrial swelling accompanied by a visible deformity of the kinetoplast. These effects were already observed after 24 h of treatment at concentrations as low as 0.5 μg/mL (Figure 4B and Figure 4C), and increased at higher concentrations of the compound (2.5 μg/mL) (Figure 4F). 

A similar mitochondrial swelling has been reported for the STL parthenolide and was attributed to effects on structural components in this organelle, as membrane proteins or enzymes [16]. Other ultrastructural alterations reported for parthenolide, helenalin and mexicanin, such as cellular or nuclear deformities [12] and effects in the distribution of subpellicular microtubules or flagella appearance were not observed for psilostachyin, suggesting that the mechanism of action of this molecule may be different. Ketoconazole has also been reported to produce mitochondrial swelling in *T. cru*z*i*, and this effect has been attributed to alterations in ergosterol metabolism [17]. 

Furthermore, psilostachyin induces deformities in the kinetoplast, suggesting that the compound could be exerting additional effects to alterations in the parasite metabolism. It is known that kinetoplast is made of a special type of DNA (k-DNA) which is located in a specialized portion of the mitochondrion. Biochemical and molecular evidences have confirmed the presence of basic proteins in the kinetoplast, indicating that histone H1- like proteins participate in the condensation of k-DNA in *T. cruzi* [18,19]. 

## 3. Experimental

### 3.1. Isolation and identification of psilostachyin

Psilostachyin (2´*R*,3a*S*,6s,8s,8a*R*)-octahydro-8-hydroxy-6,8-dimethyl-3-methylene-spiro[7H-cyclo hepta[*b*]furan-7,2´(5´H)-furan]-2,5´(3H)-dione was isolated from an organic extract [(CH_2_Cl_2_-MeOH) (1:1)] of *A. tenuifolia* and identified as previously described [14]. The purity of psilostachyin (>95%) was confirmed by high-performance liquid chromatography (HPLC).

### 3.2. Culture of Trypanosoma cruzi epimastigotes

*Trypanosoma cruzi* epimastigotes, Tulahuen strain, were grown in Diamond´s liquid medium (0.106 M NaCl, 29 mM KH_2_PO_4_, 23 mM K_2_HPO_4_, 12.5 g/L tryptose, 12.5 g/L tryptone, and 12.5 g/L yeast extract, adjusted to pH 7.2) supplemented with 10% fetal bovine serum, 7.5 μM hemin, and with 75 U/mL penicillin and 75 μg/mL streptomycin. All the experiments were carried out with the parasites at the exponential phase of growth.

### 3.3. Animals

Inbred female BALB/c mice were purchased from the Instituto Nacional de Tecnología Agropecuaria (INTA). Animals were kept according to practices described in the *Guide for the Care and Use of Laboratory Animals* of the National Research Council [20].

### 3.4. Viability and reversibility assays

*T. cruzi* epimastigotes (2 × 10^6^ parasites/mL) were incubated at 29 °C for 1-4 days in 4 mL medium (15 mL Falcon tubes), either in the absence or in the presence of psilostachyin (dissolved in DMSO) at final concentrations of 0.2–2.5 μg/mL. Aliquots of the cultures were collected every 24 h and diluted 1:10 in phosphate buffered saline (PBS) containing 2% formalin. Benznidazole (5 to 20 μM; Roche) was used as positive control. Parasites were counted in a Neubauer chamber and viability was measured by the trypan blue exclusion method [12]. 

For the reversibility assay, *T. cruzi* epimastigotes (2 × 10^6^ parasites/mL) were incubated for 3 h in the absence or in the presence of the pure compound at final concentrations of 0.2–2.5 μg/mL. Then, the parasites were centrifuged at 2,000 × g for 5 min, washed once with medium and incubated for 1 or 2 days with fresh medium free of the compound. 

### 3.5. Cytotoxicity assay

T-lymphocyte suspensions from BALB/c mice (weighing 22 ± 2 g) were employed for the determination of cell viability by the trypan blue dye exclusion method in the absence and presence of increasing concentrations of the pure compound (0.1, 1, 10 and 50 μg/mL) during 3–72 h, as previously described [14]. The selectivity index (SI) was calculated as the 50% cytotoxic concentration on murine T-lymphocytes (72 h) divided by the 50% inhibitory concentration (IC_50_) (72 h) of the compound for *T. cruzi* epimastigotes.

### 3.6. Effect of psilostachyin on T. cruzi epimastigotes in the presence of GSH

*T. cruzi* epimastigotes (2 × 10^6^ parasites/mL) were treated with 1.0 μg/mL of psilostachyin (3.6 µM) and psilostachyin plus 1-2 mM glutathione (GSH). Controls were performed with Diamond’s medium alone or with the addition of GSH. Parasite concentration was determined for 3 days by counting in a Neubauer chamber. Benznidazole (5 to 20 μM; Roche) was used as positive control.

### 3.7. Transmission electron microscopy

For electron microscopy, all procedures were carried out following Brengio *et al*. [11]. Briefly, after testing the effect of psilostachyin (0.5, 1.0 or 2.5 μg/mL) for 24 h, the epimastigotes were centrifuged at 1,000 × g for 10 min and fixed with 3% glutaraldehyde. Subsequently, they were washed three times with phosphate-buffered saline pH 7.2 (PBS) and postfixed with 2% OsO_4_ overnight. After washing twice with PBS, cells were stained with 1% uranyl acetate. The samples were dehydrated in grades of ethanol and acetone and embedded in Epon 812. Ultrathin sections were obtained using an automatic Leica-ultracut R ultramicrotome, and observed with a Siemens Elmiskop I.

## 4. Conclusions

The effect of psilostachyin, a STL isolated from *A. tenuifolia*, on *Trypanosoma cruzi* (Tulahuen) was irreversible and only partially blocked by glutathione. An important mitochondrial swelling accompanied by deformities in the kinetoplast was observed by transmission electron microscopy with this compound at a concentration as low as 0.5 µg/mL. The ultrastructural alterations may be consistent with an altered metabolism. The results shown herein, support the fact that psilostachyin probably affects key biological processes in the parasites, exerting its effects at multiple levels. Further studies are in progress for the identification of molecular targets of this compound.
